# Synthesis and anti-*Toxoplasma* activity of indole-triazole compounds on tachyzoites of RH strain

**DOI:** 10.1016/j.amsu.2022.103245

**Published:** 2022-01-08

**Authors:** Mohammad Saleh Bahreini, Aida Iraji, Najmeh Edraki, Ali Arab Monfared, Qasem Asgari

**Affiliations:** a Department of Medical Parasitology and Mycology, School of Medicine, Shiraz University of Medical Sciences, Shiraz, Iran; b Stem Cells Technology Research Center, Shiraz University of Medical Sciences, Shiraz, Iran; c Central Research Laboratory, Shiraz University of Medical Sciences, Shiraz, Iran; d Medicinal and Natural Products Chemistry Research Center, Shiraz University of Medical Sciences, Shiraz, Iran

**Keywords:** *Toxoplasma gondii*, Indole-triazole, Tachyzoites, Flow cytometry, RH strain, AIDS, Acquired immunodeficient syndrome, DMSO, Dimethyl Sulfoxide, PBS, Phosphate Buffer Solution, NTPase, Nucleoside triphosphate hydrolase, PNPase, Purine nucleoside phosphorylase, DHRF, Dihydrofolate reductase, PI, Propidium Iodide, NMR, Nuclear Magnetic Resonance, TLC, Thin layer chromatography

## Abstract

**Background:**

Conventional treatment for toxoplasmosis have severe side effects and the inability to completely eradicate the disease. Therefore, the acquisition of new anti-*Toxoplasma* drugs has always been of interest among researchers. In the present study, we prepare a new indole-triazole derivatives and evaluated their potential anti-parasitic activity against tachyzoites of *Toxoplasma* RH strain.

**Materials and methods:**

In this study, after synthesis of the two new compounds of indole-triazole, the effect of their different concentrations (2–1024 μg/ml) were determined on *Toxoplasma* tachyzoites using flow cytometry. Furthermore, tachyzoites were exposed to different concentrations of compounds (4, 16, 64, 265, 1024 μg/ml) for 1.5 h and their infectivity were evaluated in BALB/c mice.

**Results:**

The flow cytometry results indicated the benzyl derivative of indole-triazole in various concentrations had a lethal effect on tachyzoites between 11.93% and 89.66%, while the naphthalene derivative had a lethality of 26.63%–66.82%. The infectivity analysis showed that the survival time of mice at concentrations of 1024 μg/ml and 512 μg/ml of benzyl derivatives was significantly increased (*P* = 0.008 and *P* = 0.016, respectively), compared to that in the negative control group. Furthermore, survival time of mice was statistically significant at the concentration of 1024 μg/ml for naphthyl derivative (*P* = 0.012).

**Conclusion:**

Findings of the current study suggested indole triazole compounds, based on their structure and enzymes targeting, have a considerable effect on tachyzoites of *T. gondii* RH strain and can be considered as a new anti-*Toxoplasma* agent.

## Highlights

• The Benzyl and Naphtyl derivatives of Indol-triazol have dose dependently effect on tachyzoites of *T.gondii* RH strain.
• The placement of naphthyl next to indole-triazole have dose dependently effects on *Toxoplasma* tachyzoites.
• The benzyl derivative of Indole triazole showed an ability of over 80% in eliminating *Toxoplasma* tachyzoites.

## 1 Introduction

*Toxoplasma gondii* (*T. gondii*) is one of the most widespread intracellular protozoans and the agent of toxoplasmosis in humans and animals. The host of these protozoa is cats, and warm-blooded animals (birds and mammals) are considered intermediate hosts [1,2]. Toxoplasmosis in people with a normal immune system is usually benign with self-limiting adenopathy. However, infection in people with immunodeficiency is dangerous and may represent toxoplasmic encephalitis, which may be fatal [3]. Common drugs (pyrimethamine + sulfadiazine) as first-class drugs for the treatment of toxoplasmosis. Frequent toxic side effects including suppression of bone marrow function [4,5], hematologic toxicity, teratogenic and renal complications are the major limitations for the use of these drugs [[6], [7], [8]]. Therefore, the introduction of effective drugs with minimal side effects seems necessary. Recently, promising results have been obtained regarding new compounds against *T. gondii* by targeting parasitic enzymes, such as nucleoside triphosphate hydrolase (NTPase) [9], purine nucleoside phosphorylase (PNPase) [10], adenosine kinase [11], dihydrofolate reductase (DHRF) [12], and calmodulin-domain protein kinase [13]. Indole derivatives theoretically inhibit NTPase in both tachyzoite and bradyzoite forms of *T. gondii* [14]. Moreover, it has been shown that triazole compounds cause parasite death by inhibiting the PNPase enzyme and reducing purine bases [15]. In this regard, the present study aimed to prepare indole triazole derivatives and test their efficiency against tachyzoites of *T. gondii* RH strain.

## 2 Methods

### 2.1 Synthetase of derivatives

**Fig. 1:**
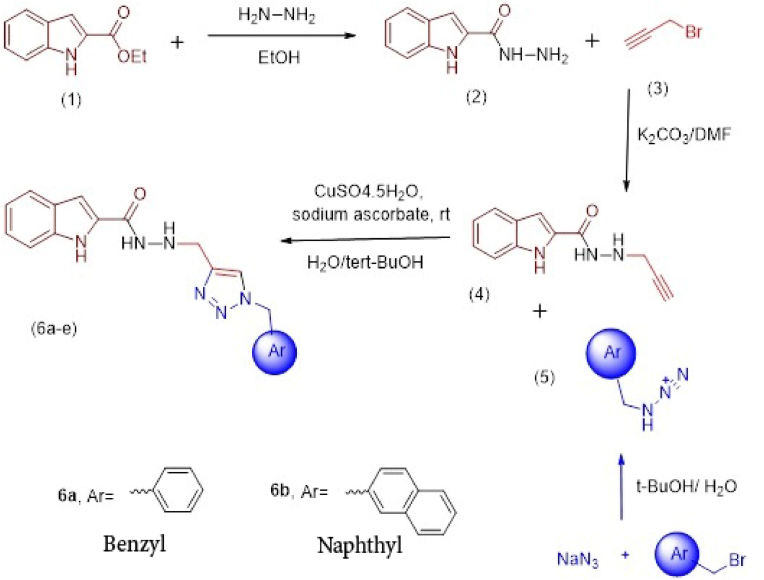
Protocol, reagents, and conditions for the synthesis of derivatives: The reaction of ethyl 1H-indole-2-carboxylate (**1**) and hydrazine hydrate in refluxing ethanol gave compound **2**. In the next step, the reaction of compound **2** and 3-bromoprop-1-yne (**3**) in the presence of K_2_CO_3_ in DMF at 80 °C resulted in the desired N'-(prop-2-yn-1-yl)-1H-indole-2-carbohydrazide **(4)**. Different alkyl or aryl halides were reacted with sodium azide in the presence of triethylamine in *tert*-BuOH/H_2_O. After around 30 min, intermediate **4** were added to the mixture of reaction in the presence of catalytic amount of CuSO_4_5H_2_O (7 mol%) sodium ascorbates (20 mol%) to give the corresponding products **6a, b.**

After optimizing the structure of the compounds and designing the best inhibitor, the number of derivatives of the strongest compounds that have the lowest binding energy was synthesized and purified using appropriate methods [16]. All chemicals and reagents were purchased from Sigma-Aldrich. Melting points were determined using Kofler hot stage apparatus. Nuclear Magnetic Resonance (NMR) spectroscopy were recorded on a Bruker 300 spectrometer and chemical shifts were expressed as *δ* (ppm). Synthesis of the *N'-((1-benzyl-1H-1,2,3-triazol-*4-yl*) methyl)-1H-indole-2-carbohydrazide* (compounds A) and *N'-((1-(naphthalen-2-ylmethyl)-1H-1,2,3-triazol-*4-yl*) methyl)-1H-indole-2-carbohydrazide*(compounds B) was conducted according to the steps shown in Fig. 1.

#### 2.1.1 Synthesis of 1H-indole-2-carbohydrazide (2)

One mmol of ethyl 1H-indole-2-carboxylate 1 was dissolved in ethanol in presence of the catalytic amount of acetic acid and the mixture was stirred at room temperature for 30 min. Then, hydrazine hydrate (3 mmol) was added and the reaction was continued for 12 h under reflux conditions. After completion of the reaction (thin layer chromatography (TLC) control), the participants were collected and recrystallized in ethanol. Yield: 88%, ^1^HNMR (300 MHz, DMSO-*d*_*6*_): 11.63 (s, 1H, NH), 9.80 (s, 1H, NH indole), 7.59 (d, *J* = 7.5 Hz, 1H, Ar), 7.43 (d, *J* = 7.5 Hz, 1H, Ar), 7.19–7.00 (m, 3H, Ar), 4.55 (brs, 2H, NH_2_) ppm.

#### 2.1.2 Synthesis of N'-(prop-2-yn-1-yl)-1H-indole-2-carbohydrazide (4)

In the current study, 1H-indole-2-carbohydrazide (compound **2**, 1 mmol), 3-bromoprop-1-yne (compound **3**, 4 mmol) and K_2_CO_3_ (0.6 mmol) were dissolved in N-dimethylformamide (DMF) (30 mL). The reaction mixture was mixed at 70 °C for 24 h. After completion of the reaction (control), it was cooled to room temperature, extracted by ethyl acetate, and washed three times using water. It was then dried over anhydrous sodium sulfate and the solvent was evaporated under vacuum. Yield: 43%, ^1^HNMR (300 MHz, Acetone-*d*_*6*_): 10.98 (s, 1H, NH), 8.97 (s, 1H, NH indole), 7.67–7.58 (m, 2H, Ar), 7.28–7.23 (m, 2H, Ar), 7.07 (t, *J* = 7.0 Hz, 1H, Ar), 3.96 (brs, 3H, CH_2_ and NH), 2.831 (s, 1H, CH) ppm. ^13^CNMR (75 MHz, Acetone-*d*_*6*_): 160.39, 136.87, 129.89, 127.74, 123.97, 121.82, 120.08, 112.28, 102.97, 78.20, 74.39, 44.36 ppm.

#### 2.1.3 General method for synthesis the substituted derivative of N'-((1-benzyl-1H-1,2,3-triazol-4-yl) methyl)-1H-indole-2-carbohydrazide

The final step was performed by the click reaction of compound **4** and *in situ* prepared azides **5**. For this purpose, a solution of aryl halide (1.1 mmol), sodium azide (0.06 g, 0.9 mmol), and triethylamine (0.13 g, 1.3 mmol) in water (4 mL) and *tert*-butyl alcohol (4 mL) was stirred at room temperature for 30 min. Then, compound 4 (0.5 mmol) and CuSO_4_·5H_2_O (7 mol%) sodium ascorbates (20 mol%) was added to the mixture and it was continued for 24–48 h The mixture was diluted with water, extracted with chloroform, and dried over anhydrous Na_2_SO_4_. After evaporation of the solvent, the residue was recrystallized from EtOH to give a pure product. In the case of some compounds, they were purified using plate chromatography with ethyl acetate as eluent.

### 2.2 Animal subjects

In the current study, 4-6 week-inbred BALB/c female mice (weight 25–30 gr) were obtained from Center of Comparative and Experimental Medicine Shiraz University of Medical Sciences, Shiraz, Iran. The animals were kept at 22 °C and 40–50% relative humidity and had access to standard food and water ad libitum. During the experiments, animals were housed in cages and maintained under controlled conditions. All stages of this study include the selection of specific animal species, the minimum animal required for statistical and true research accuracy, maintenance, permissible injection rate, minimal harassment at various stages of the investigation and ethical death of animals were undertaken based on guidelines for laboratory animals and Ethical Committee of Shiraz University of Medical Sciences [17].

### 2.3 Preparation of the tachyzoites of Toxoplasma gondii RH strains

RH strain of *T. gondii*, a gift from Professor Ahmad Daryani (Toxoplasmosis Research Center, Mazandaran University of Medical Sciences, Sari, Iran), was injected intraperitoneally (IP) to 10 BALB/c mice. After 4 days, the mice were killed by ethical standards. Then, a longitudinal incision was made in the abdomen on the skin, the skins were removed, the peritoneal area was flushed with normal saline, the most tachyzoites were collected from the peritoneal area. Based on previous study the yielded tachyzoites were separated and purified [18].

### 2.4 Investigation of the direct effect of indole-triazole drives on tachyzoites using flow cytometry

The indole triazole drives were dissolved in DMSO and PBS was used to prepare a dilution series of these materials from 2 to 1024 μg/ml. In this dilution series, the DMSO concentration was not more than 1%. Then 2 × 10^5^ tachyzoites were counted using a hemocytometer slide and placed in each Eppendorf tube; 15–20 μl of tachyzoites from the previous step was added between the hemocytometer and cover glass, the number of tachyzoites was counted in all four outer squares divided by four (the mean number of cells/square). The number of tachyzoites per square x 10^4^ = the number of tachyzoites/ml of suspension. Thus, 2 × 10^5^ tachyzoites in a volume of 500 μl were estimated. Then, the tachyzoites were placed near 500 μl of the prepared concentrations of compounds at room temperature for 3 h. After this period, propidium iodide (PI) with a final concentration of 50 μg/ml was added and incubated for half an hour in the dark. Saponin 0.2% was used for positive control. These tubes were placed inside the FACS Calibur flow cytometer to investigate the effect of derivatives and controls. Evaluation of parasite death based on staining and fluorescence of this dye was examined by flow cytometer.

### 2.5 Infectivity assessment of tachyzoites exposed to indole-triazole drives *in vivo*

Tachyzoite of RH strain collected from the peritoneum of infected mice were separated by different centrifugation cycles and washed with PBS [18]. Then, 10^5^ tachyzoites were exposed to various concentrations of compounds (4, 16, 64, 265, 1024 μg/ml) for 1.5 h and 100 μL of each concentration was injected IP to 10 BALB/c mice. The mice were monitored daily and the time of death was recorded.

### 2.6 Statistical analysis

Statistical analysis was carried out using SPSS Software version 16 (IBM Corporation, Armonk, NY, USA). Statistical differences between the test and control groups were analyzed using one-way analysis of variance (ANOVA) with a confidence interval of 95%. A *P* < 0.05 was considered statistically significant.

## 3 Results

### 3.1 Flow cytometry results

**Fig. 2:**
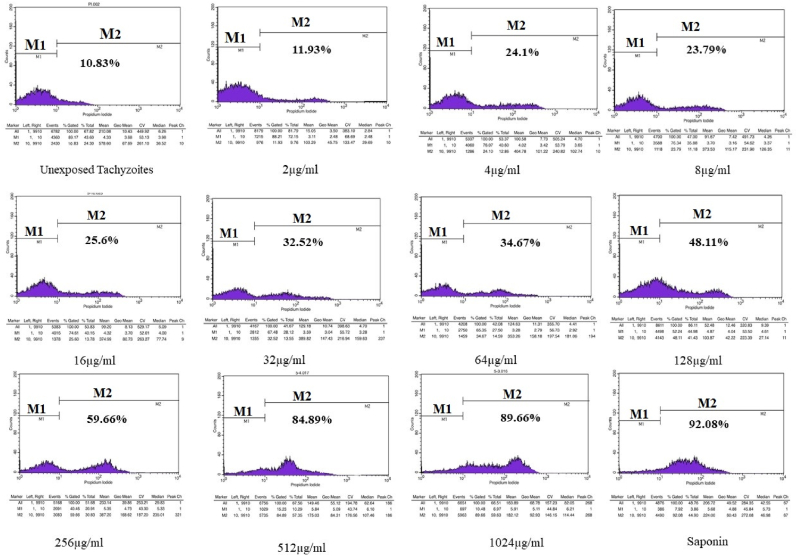
The result of flow cytometry tests on tachyzoite of *Toxoplasma* exposed to, saponin as positive control and different doses of compound A. The PI dye stains DNA of dead parasites. The area of the colored part in M2 indicates the percentage of dead tachyzoites. As much as the color area shift to M2, t more tachyzoites die from exposure to the compound. (For interpretation of the references to color in this figure legend, the reader is referred to the Web version of this article.)

**Fig. 3:**
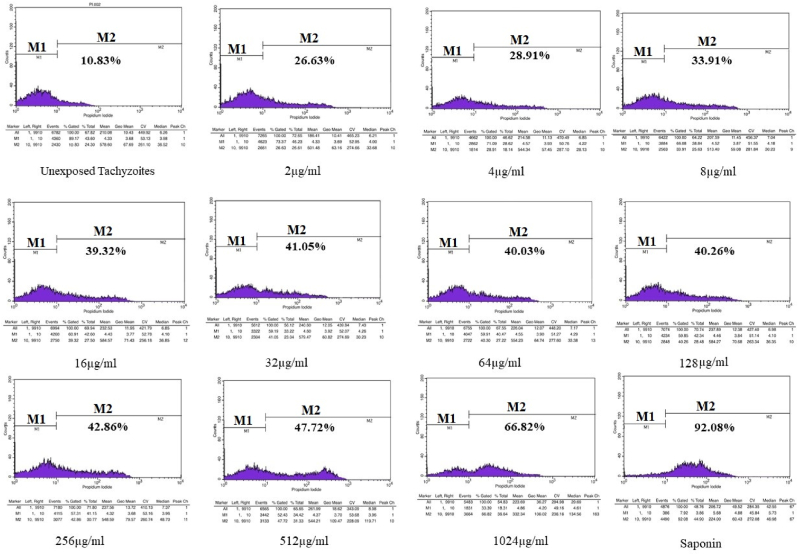
The result of flow cytometry tests on tachyzoite of *Toxoplasma* exposed to, saponin as positive control and different doses of compound B. The PI dye stains DNA of dead parasites. The area of the colored part in M2 indicates the percentage of dead tachyzoites. As much as the color area shift to M2, t more tachyzoites die from exposure to the compound. (For interpretation of the references to color in this figure legend, the reader is referred to the Web version of this article.)

Based on the flow cytometry results, 10.83% of the *Toxoplasma* tachyzoites collected from peritoneal passages was survived (unexposed group). Apoptosis or mortality was seen in 92.08% of tachyzoites that were exposed to 0.2% saponin using PI stained (Fig. 2). The flow cytometry results indicated the benzyl derivative of indole-triazole in various concentrations had a lethal effect on tachyzoites between 11.93% and 89.66%, while the naphthalene derivative had a lethality of 26.63%–66.82% (Fig. 3, 4). Flow cytometry analyses of various concentration (2–1024 μg/ml) of compound A and compound B on *Toxoplasma* tachyzoites viability was exhibited in Figs. 3 and 4. The IC_50_ of compound A and compound B were 157.8 μg/ml and 459.6 μg/ml, respectively.

### 3.2 The result of infectivity test

The result of infectivity test revealed that all mice died after 17 days. The survival time of mice was directly associated with the compound's concentration. Moreover, the number of live mice increased when the concentration of the compound raised (Table 1). At concentrations of 1024 μg/ml and 512 μg/ml of benzyl derivative, the survival time of mice was significantly increased (*P* = 0.008 and *P* = 0.016, respectively), compared to that in the negative control group. Furthermore, survival time of mice was statistically significant at the concentration of 1024 μg/ml for naphthyl derivative (*P* = 0.012).

**Table 1:** The mean of life duration (days) of mice groups inoculated by *Toxoplasma* tachyzoites exposed to different concentration of compound A and B.

concentrations	4 μg/ml		16 μg/ml		64 μg/ml		256 μg/ml		1024 μg/ml		Control
Compound	A	B	A	B	A	B	A	B	A	B	
**Longevity time mean of mice (days)**	5.6	5.7	5.8	7.3	7.1	7.5	10.2	7.8	15.2	12.3	5.1
**Total number of mice (60)**	**10**		**10**		**10**		**10**		**10**		**10**

## 4 Discussion

Treatment of toxoplasmosis is difficult due to the ineffectiveness and side effects of existing drugs and the fact that re-infection may occur rapidly [19]. Therefore, introducing new anti-*Toxoplasma* drugs seems essential. Several studies have turned to the use of anti-*Toxoplasma* drugs for better efficacy, which are structurally similar to the parasite's essential substances for life and by targeting parasite enzymes [[9], [10], [11]]. NTPase is an essential enzyme for the rapid proliferation of tachyzoites within the parasitophorous vacuole, which is secreted by the parasite into the vacuole. Therefore, this enzyme is considered as a drug target. Indole derivatives inhibit NTPase and due to their structural similarity to tryptophan interfere with the absorption and metabolism of this essential amino acid of the parasite [14]. Tryptophan, as one of the indole compounds, is essential for *Toxoplasma* and receives it from the host cell. Stimulation of the indoleamine-2,3-dioxygenase pathway in host cells degrades tryptophan and deprives the parasite, causing a range of inhibition of parasite growth in fibroblast, epithelial, and endothelial cells of animal species, including humans [20]. In the study of Takashi et al., [14] the highest inhibition of NTPase and consequent parasite death in the form of tachyzoite was specific to the indole compound. In this study, by working on substitutions of this compound, more effective materials were obtained.

Studies on azole compounds have also shown that these compounds have a very effective role against *Toxoplasma* parasites [21]. In the study of Erica et al., the effect of fluconazole (with two triazole rings) and itraconazole (with two triazole rings and diazole rings) were assessed, and it was shown that these compounds can be effective and safe drugs for the treatment of *Toxoplasma* [22]. Katarzyna et al. investigated the effect of triazole-based compounds on *Toxoplasma* in mouse fibroblast culture, and the results showed triazole-based compounds 4 times more effective than sulfadiazine in addition to low toxicity to the host. Moreover, in this study, it was shown that the PNPase is most likely the drug target of triazole compounds [15]. This enzyme is an essential substance in the production of de novo purine nucleotides and ribose phosphate strands [23]. Triazole-based compounds inhibit the PNPase enzyme and thus reduce purine bases and cause parasite death [24].

The results of the current study demonstrated that the placement of naphthyl next to indole-triazole (compound A) has dose-dependently effects on *Toxoplasma* tachyzoites, as Asgari et al., have shown in their study with evaluating the direct effect of two new Naphthalene-Sulfonyl-Indole compounds [25]. However, this effect was greater in benzyl compounds (A) than in naphthalene compound (B), which may be due to its smaller structure and thus greater penetration into the parasite. Also, in various studies, the role and effect of benzyl-containing compounds on various microorganisms and parasites have been confirmed [[26], [27], [28], [29]], as in our study, the benzyl derivative showed an ability of over 80% in eliminating *Toxoplasma* tachyzoites. The main proposed mechanism of action by benzyl compounds may be impaired macromolecular biosynthesis and cell death. Suppression of polyamines and impact on the calmodulin by compounds containing benzyl may be another factor in reducing parasite growth [30,31]. The present study only was examined the direct effect of the compounds in the *in vitro*. In future studies, the effect of compounds *in vivo* and evaluation of their toxicity on cells by MTT assay as well as evaluation of toxicity in mice by examining blood factors and tissue changes can be done.

## 5 Conclusion

The results of this study showed that indole triazole-based formulations, due to their similarity to the essential substances of the parasite and their effect on its essential enzymes, have a high lethality effect against tachyzoites of *T. gondii* RH strain and can be suitable candidate for further research into an effective toxoplasmosis drug.

## Ethical approval

The Ethics Committee of Shiraz University of Medical Sciences gave approval for this study (IR.SUMS.REC.1398.678).

## Sources of funding

The author(s) received no financial support for the research, authorship and/or publication of this article.

## Funding

This research was funded by the project 97-01-01-18897 from 10.13039/501100004320Shiraz University of Medical Sciences, Shiraz, Iran.

## Author contribution

QA and MSB Conceived and designed the experiments. MSB, AI, NE, and AAM: Performed the experiments. MSB, QA, and AI: Analyzed and interpreted the data. QA: Contributed reagents, materials, analysis tools or data. QA, MSB, AI: Writing - original draft, Writing - review & editing. All authors read and approved the final manuscript.

## Registration of research studies

1. Name of the registry: N/A.

2. Unique Identifying number or registration ID: N/A.

3. Hyperlink to your specific registration (must be publicly accessible and will be checked): N/A.

## Availability of data and materials

The dataset used and/or analyzed during the current study is available from the corresponding author upon reasonable request.

## Consent

Written informed consent was obtained from the patients for publication of this case report and accompanying images. A copy of the written consent is available for review by the editor-in-chief of this journal on request.

## Provenance and peer review

Not commissioned, externally peer-reviewed.

## Guarantor

**Dr.** Qasem Asgari, Department of Parasitology and Mycology, School of Medicine, Shiraz University of Medical Sciences, Shiraz, Iran. Tel/Fax: +98-71-32305291; Email: asgarig@sums.ac.ir.

## Declaration of competing interest

The authors declared no potential conflicts of interests.
