# Supplementary material for: Synthesis and anti-Toxoplasma activity of indole-triazole compounds on tachyzoites of RH strain
Source: Ann Med Surg (Lond). 2022 Jan 8;74:103245. doi: 10.1016/j.amsu.2022.103245 (PMC8777237; doi:10.1016/j.amsu.2022.103245)
Supplement: Multimedia component 1 [file mmc1.doc]

**The ARRIVE Guidelines**

**Animal Research: Reporting *In Vivo* Experiments**

|  | **Item** | **Recommendation** |
| --- | --- | --- |
| **TITLE:** | **Checked** | **Synthesis and anti-Toxoplasma activity of indole-triazole compounds on tachyzoites of RH strain** |
| **ABSTRACT:** | **Checked** | Page1 |
| **INTRODUCTION** | **Checked** | **Page2** |
| - **Background** | **Checked** | Page2 |
| - **Objectives** | **Checked** | Page2 |
| **METHODS** | **Checked** | Page2-6 |
| - **Ethical statement** | **Checked** | Page 9 line 202-204. |
| - **Study design** | **Checked** | N/A |
| - **Experimental procedures** | **Checked** | Page 4. line: 92-93 |
| - **Experimental animals** | **Checked** | Page 5. line: 111-115 |
| - **Housing and husbandry** | **Checked** | Page 4. line: 88-93 |
| - **Sample size** | **Checked** | N/A |
| - **Allocating animals to experimental groups** | **Checked** | Page 5. line: 111-115 |
| - **Experimental outcomes** | **Checked** | Page 5. line: 111-115 |
| - **Statistical methods** | **Checked** | Page 6. line: 117-120 |
| **RESULTS** | **Checked** | Page 6. line: 123-130 |
| - **Baseline data** | **Checked** | Page 6. line: 123-130 |
| - **Numbers analyzed** | **Checked** | Page 6. line: 123-130 |
| - **Outcomes and estimation** | **Checked** | Page 6. line: 123-130 |
| - **Adverse events** | **Checked** | **N/A** |
| **DISCUSSION** | **Checked** | Page 7,8 |
| - **Interpretation/scientific implications** | **Checked** | Page 7,8 |
| - **Generalisability/translation** | **Checked** | **N/A** |
| - **Funding** | **Checked** | Page 7,8 line 197-198 |
